# Effects of Antihypertensive Drugs on Cognitive Function in Elderly Patients with Hypertension: A Review

**DOI:** 10.14336/AD.2020.1111

**Published:** 2021-06-01

**Authors:** Wei Yang, Hongyu Luo, Yixin Ma, Sicong Si, Huan Zhao

**Affiliations:** Department of Geriatric Medicine, Xuan Wu Hospital, Capital Medical University, Beijing 100053, China

**Keywords:** hypertension, elderly, cognitive decline, antihypertensives

## Abstract

Hypertension is a common comorbidity that contributes to the development of various cardiovascular disorders in elderly patients. Moreover, hypertension has been associated with cognitive decline and dementia. Cognitive impairment leads to increased morbidity and mortality in elderly patients with hypertension. However, previous studies investigating the association between blood pressure (BP), BP variability (BPV), and antihypertensive drug use and the risk of cognitive impairment in elderly patients with hypertension have reported inconsistent findings. Given the global burden of hypertension, the aging population, and the low quality of life associated with cognitive impairment, a more comprehensive understanding of the association between hypertension and cognitive decline is needed. In this review, we summarized the current preclinical evidence and clinical research regarding the association of BP control, BPV, and antihypertensive drug use and cognitive function. We particularly focused on the differences among categories of antihypertensive drugs. We concluded that the correlation of BP and risk of cognitive function is non-linear and dependent on a patient’s age. Intensive BP control is generally not recommended, particularly for the oldest-old. Increased BPV and characteristics of orthostatic hypotension in the elderly also increase the risk of cognitive decline. The current evidence does not support one category of antihypertensive drugs as superior to others for preventing dementia in elderly patients with hypertension.

Hypertension is a common morbidity and a major risk factor for cardiovascular disease and stroke, particularly in the elderly. The China Patient-Centered Evaluative Assessment of Cardiac Events (PEACE) Million Persons Project (2014 - 2017) showed that nearly half of the participants aged 35 - 75 years had hypertension, and the prevalence increased with age [1]. A large-scale meta-analysis including 242 studies from 45 countries showed a hypertension prevalence of 53 - 76% in people over 65 years of age in low- and middle-income countries [2]. Effective blood pressure (BP) management with antihypertensive drugs can substantially reduce the incidence of cardiovascular diseases in these patients [3].

Cognitive impairment and subsequent dementia are significant causes of morbidity and mortality among the elderly [4]. In China, the prevalence of cognitive impairment in people over 60 years of age is 10.12% [5]. Accumulating evidence has demonstrated that hypertension is an important risk factor for cognitive impairment in the elderly [6, 7]. Increased BP variability (BPV), an important feature of BP, has also been associated with increased risk of dementia [8]. Recent meta-analyses showed that any antihypertensive drug that effectively reduces BP can also reduce the risk of cognitive decline, dementia, and Alzheimer's disease (AD) in patients with hypertension [9, 10]. However, it remains unknows if the benefits of BP reduction on cognitive function are similar among antihypertensive. It is also unclear if there is a certain threshold for lowering BP that would favorably impact cognitive function, particularly for the elderly.

Although considerable observational studies, clinical trials, and systematic reviews have shown that antihypertensive drugs may be associated with reducing cognitive impairment, the results of these reports vary significantly [9, 10]. The inconsistent results of these studies may be explained by differences in antihypertensive medications, different BP lowering targets, and the influence of these treatments on BPV in the elderly. Currently, five categories of antihypertensive drugs are used to treat elderly patients with hypertension. These include angiotensin converting enzyme inhibitors (ACEIs), angiotensin II receptor blockers (ARBs), β-blockers, calcium channel blockers (CCBs), and diuretics [11]. Recent efforts have been made to understand the potential differences of these antihypertensive drugs on cognitive function. We aimed to summarize the threshold of BP control, BPV, and antihypertensive drug use and cognitive function in recent years, with a particular focus on potential differences among antihypertensive drugs.

## Hypertension treatment, target BP, and cognitive function in the elderly with hypertension

The diagnostic criteria for elderly patients with hypertension vary according to established guidelines in different countries. According to the 2019 Chinese Guideline for the Management of Hypertension in the Elderly, for those aged > 65 years with no use of antihypertensive drugs, hypertension is defined as systolic blood pressure (SBP) ≥ 140 mmHg and/or diastolic blood pressure (DBP) ≥ 90 mmHg based on the average of three daily recordings [11]. For those who had a previous diagnosis of hypertension and were treated with antihypertensive drugs, hypertension was diagnosed even if the BP < 140/90 mmHg. The grade and risk stratification of hypertension in the elderly is applied with the same criteria as that in the general adult population [11]. This guideline provides recommendations on the timing for initiating antihypertensive treatments in the elderly. Specifically, for those aged 65 ~ 80 years, a combined management of therapeutic lifestyle change (TLC) and antihypertensive drug therapy should be initiated if BP ≥ 140/90 mmHg and to maintain BP < 140/90 mmHg. For those aged > 80 years, antihypertensive drug therapy should be initiated if BP ≥ 150/90 mmHg and to maintain BP < 150/90 mmHg. If well tolerated, the subsequent goal is to maintain BP < 140/90 mmHg. For those with fragility and aged > 80 years, antihypertensive drug therapy should be initiated if BP ≥ 160/90 mmHg and to maintain SBP of 130 ~ 150 mmHg. Antihypertensive drug therapy should not be discontinued if the therapy is well tolerated.

For elderly individuals with hypertension, cognitive impairment mainly manifests as disorders of executive function, memory, motor speed, attention, and other cognitive degrees of damage [12]. Many studies have shown that a long-term increase in BP can lead to cognitive impairment. However, the relationship between hypertension and cognitive impairment differs based on age [13]. A long-term follow-up study showed that the relationship between BP and cognitive function was U-shaped [14]. Low SBP or DBP may also increase the risk of cognitive impairment in the elderly > 80 years of age. The SBP of dementia subjects was significantly lower than that of non-dementia subjects. There are few long-term cohort studies on BP and cognitive impairment in hypertensive elderly patients aged > 85 years. Data from the Leiden 85-plus Study showed that lower SBP in the oldest-old taking antihypertensive drugs was associated with higher mortality and faster decline in cognitive function [15]. In another cohort of hypertensive elderly patients aged > 85 years, higher BP was related to better MMSE scores at a 5-year follow-up analysis [16]. It is worth noting that cognitive dysfunction associated with hypertension may not be limited to vascular dementia, as it may also be associated with dementia related to other causes. For vascular dementia, hypotension was associated with reduced cerebral blood perfusion, while long-term hypertension was associated with atherosclerosis of small cerebral vessels, both of which have been associated with risks of vascular dementia. From an empirical perspective, the protective effect of antihypertensive drugs on target organs is remarkable. In addition, the antihypertensive medications themselves may improve cognition. However, there is still a lack of large-scale clinical studies to support this.

Studies evaluating the association between BP target and cognitive function in the elderly present inconsistent results. An early study including 2,977 patients with type 2 diabetes mellitus (T2DM, mean age 62 years) showed that intensive BP control (target SBP < 120mmHg) compared with standard BP control (target SBP < 140 mmHg) did not produce a measurable effect on cognitive decline at 40 months of follow-up [17]. The recently published SPRINT MIND study included 9,361 subjects (mean age 67.9 years) and showed that, compared to the treatment target of SBP < 140 mmHg, intensive BP control of SBP < 120 mmHg did not significantly reduce the risk of dementia [18]. However, the authors acknowledged that their findings may not have been statistically adequate, as the study ended prematurely with fewer cases of dementia than expected. Interestingly, a recently published secondary analysis of the Systolic Blood Pressure Intervention Trial showed that for patients aged ≥ 80?years with hypertension and no T2DM, intensive SBP control of < 120 mmHg compared to standard SBP control < 140 mmHg lowered the risk of major cardiovascular events, mild cognitive impairment (MCI), and death, with increased risk of changes in kidney function [19]. However, no incidence of dementia was observed. Although MCI significantly increases the risk of dementia, this progression is uncertain and patients with MCI may return to normal cognition if appropriately managed. Moreover, differences in antihypertensive drugs, comorbidities (T2DM), and follow-up strategies may contribute to inconsistent findings and should be considered in future studies.

## BPV and cognitive function in the elderly with hypertension

Elderly patients with hypertension are vulnerable to BP fluctuation, which is referred to as BPV. An increasing number of studies indicate that BPV plays an important role in the development of target organ injury and cardiovascular events, which is likely independent of absolute BP. Observational studies based on the results of a few months or years of ambulatory BP assessment have generally shown that increases in BPV are significantly related to a high risk of cognitive impairment and dementia. A previous study aimed to clarify the relationship between daily BPV based on familial BP and the risk of dementia and its subtype development in a prospective study of an elderly Japanese population [8]. This study showed that increases in daily BPV were significantly associated with the occurrence of all-cause dementia, even after adjusting for BP and other potential risk factors for dementia. These findings suggest that BPV is an important indicator of dementia development, and a possible target for intervention [8]. Another study observed the relationship between long-term BPV and cognitive impairment in elderly patients with hypertension, and showed that a large fluctuation in long-term BP (26 year follow-up) was related to a long-term risk of dementia [20]. Moreover, the long-term risk of dementia correlated with the extent of BP increase or decrease, rather than the direction of BP changes [20]. A recent meta-analysis by Phillip *et al*, showed that an increase in BPV was related to an increase in the incidence of cerebral small-vessel disease (CSVD) [21]. The mean value of BPV in patients with CSVD was generally higher than that in non-CSVD patients regardless of mean BP levels [22]. Variations in SBP may damage the brain microvasculature, leading to cerebral atrophy and cerebrovascular diseases, ultimately causing dementia [22]. The hemodynamic instability caused by the increase in BPV may increase shear stress, which directly leads to small vessel disease, cerebral hypoperfusion, and subsequent neuronal damage [22].

## Orthostatic hypotension and cognitive function in the elderly with hypertension

Orthostatic hypotension (OH) refers to a decrease in BP when moving from a supine position to an upright position, often accompanied by drowsiness, vertigo, blurred vision, and other symptoms of cerebral perfusion insufficiency, representative of postural BP change [23]. About 5 - 30% of elderly individuals have OH, and people with OH have a higher risk of cardiovascular disease and mortality [23, 24]. OH has also been associated with cognitive decline in recent studies.

Collaby *et al*, [1] suggested that a decrease in SBP in an upright position might cause high signal intensity in the deep white brain matter [25]. Recent studies also reported that OH is an independent risk factor for ischemic stroke, which increases the incidence rate of AD [26]. With increases in age, the possibility of cognitive dysfunction in elderly patients increases [26]. Various mechanisms may explain the relationship between cognitive dysfunction and OH. Brain regions responsible for cognitive function may be involved in regulating cardiovascular activities. Therefore, neurodegeneration of these brain regions may lead to cognitive impairment and OH [26]. OH can lead to insufficient frontal lobe perfusion, which may alter executive function [27]. A decrease in cerebral blood flow may also lead to subcortical infarction and the formation of tension around the white matter; amyloid deposition may be involved in this process, as ischemic stress is related to amyloid deposition in humans [27]. Finally, cerebral hypoperfusion caused by long-term BP changes in OH patients may cause ischemic demyelination, which is the cause of cognitive dysfunction [27]. Rebecca *et al*, performed a systematic review and meta-analysis of eight longitudinal studies regarding the association between OH and cognitive function [28]. Using MMSE as the main cognitive assessment scale, they found no significant association between OH and cognitive impairment in four of the studies with a short follow-up time (1 - 4 years) [28]. However, a significant correlation between OH and cognitive decline was observed in long-term follow-up (3 - 25 years) studies that used a detailed cognitive assessment scale [28]. Moreover, nearly 2/3 of elderly patients with OH and dementia have no symptoms related to OH [28]. This highlights the importance of screening for OH in the elderly regardless of their symptoms.

## Antihypertensive drugs and cognitive function in the elderly with hypertension

**Figure 1.**
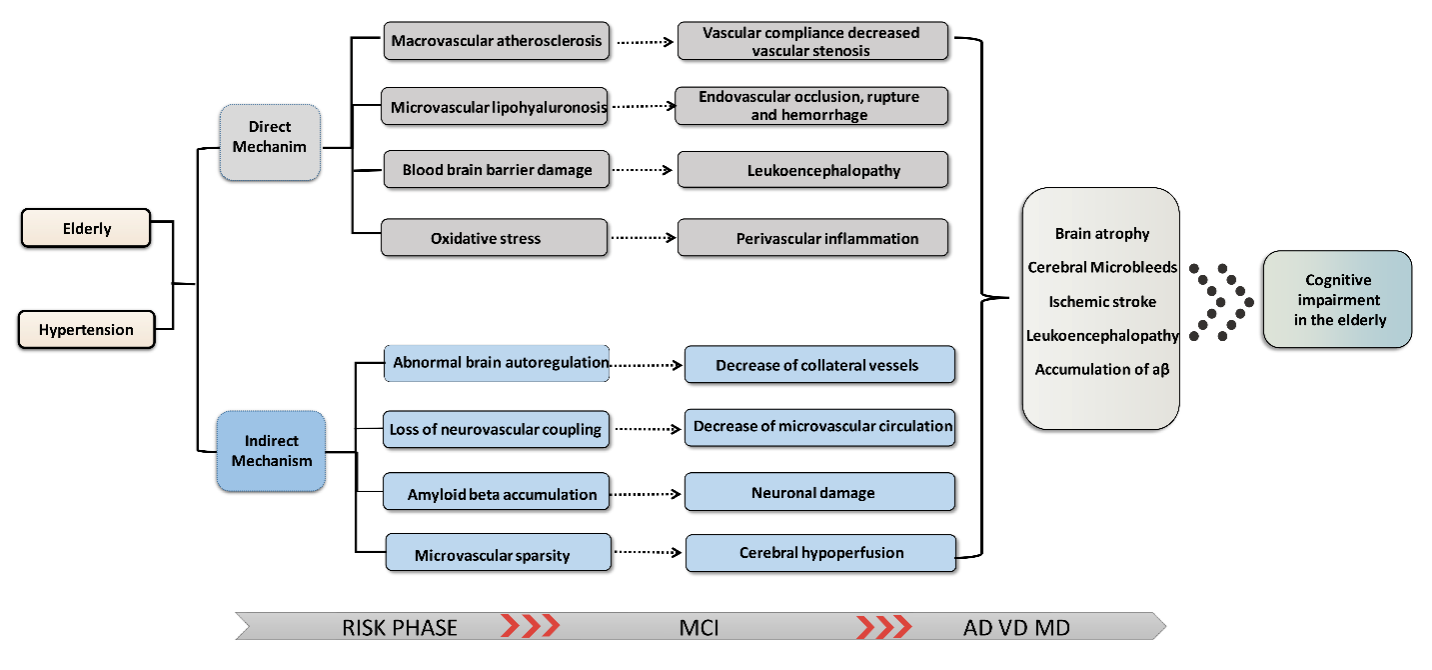
The underlying mechanism of cognitive impairment caused by hypertension in the elderly.

Multiple mechanisms have been proposed to underlie the association between hypertension and cognitive decline in the elderly (summarized in Fig. 1). Lowering BP with antihypertensive drugs may exert potential cognitive benefits in the elderly via the mechanisms described below. Some category-specific mechanisms are also described for each antihypertensive drug in this setting (Table 1).

## Angiotensin converting enzyme inhibitors (ACEIs)

Genetic and pathological studies show that ACEs are related to AD [29]. Preclinical studies show that a variety of proteases are involved in the catabolism of amyloid β-protein (Aβ) [30]. ACE can convert Aβ 42, the main component of amyloid plaques in AD, into Aβ 40, the main component of amyloid angiopathy in the brain [30]. In addition, high levels of angiotensin II (Ang II), resulting from ACE mediated conversion of Ang I, cause cerebral vasoconstriction and injury, increasing the risk of AD [30]. However, the exact molecular pathway via which ACE participates in the pathogenesis of AD is not clear.

**Table 1:** Representative studies evaluating the effects of antihypertensives on the cognitive function.

	Study and main findings	References
ACEIs	PROGRESS: perindopril reduced vascular cognitive impairment in patients with hypertension	31, 32
	Ohruit et al: perindopril improved MMSE scores	34
	Qiu et al: ACEIs reduced risk of AD in the absence of ApoE4, but not in ApoE4 carriers	37
Diuretics	Cache Country Cohort Study: thiazides and potassium sparing diuretics reduced AD risks	39
	Peters et al meta-analysis: no association of diuretics and cognitive impairment in elderly > 65 y	10
ARBs	SCOPE: candesartan improved MMSE scores	47
	OSCAR: eprosartan improved MMSE scores	48
CCBs	syst EUR: nitrendipine decreased risk of AD in patients > 60 y without previous stroke	58
	Angeli et al meta-analysis: dihydropyridine has benefit on cognitive function in stroke patients,	65
β-blockers	Yasar et al: β-blockers can reduce the risk of AD in the elderly	76
	Khachaturian et al: β-blockers alone can reduce the risk of cognitive decline	77
	Gelber et al: β-blockers had little effect on cognitive improvement in elderly patients with hypertension	78

In addition to controlling BP, ACEIs have been proposed to play a role in the development and progression of AD in the elderly due to their effects on Aβ clearance in the brain [29]. Some previous studies showed that ACEIs can delay the decline of cognitive function in hypertensive elderly patients with MCI by improving cerebral blood flow. In the PROGRESS study, the ACEI perindopril reduced the risk of vascular cognitive impairment in patients with hypertension [31, 32]. Moreover, ACEIs can slow the rate of cognitive decline and reduce the conversion rate of Aβ in patients with hypertension and AD [32]. The ability of individual ACEIs to pass through the blood-brain barrier (BBB) may affect their efficacy. Yamada K *et al*, showed that different types of ACEIs have varying effects on cognitive impairment, and that perindopril can significantly improve cognitive impairment via passing the BBB and stimulating secretion of extracellular acetylcholine [33]. Consistently, Ohruit *et al*, found that perindopril use was associated with improved MMSE scores [34], suggesting that central ACEIs may benefit cognitive function in patients with hypertension.

Other studies have yielded contradictory results. In an AD animal model using APP transgenic mice, ACEI treatment either had no effect or accelerated the development of AD, which was dependent on the duration of drug use [35]. Long-term (6 months) use of captopril increased Aβ deposition in the brain, while short-term (1 month) use did not change Aβ protein and plaque levels [36]. Another cross-sectional study showed that the effect of ACEIs on AD in the elderly vary according to APOE genotype [37]. Use of ACEIs was associated with a reduced risk of AD in the absence of ApoE4, but no such effect was observed in those carrying the ApoE4 allele [37]. The possible mechanism of the interaction between ApoE4 and ACEIs on AD is not clear. It is possible that when ApoE4 is present, the effect of ACEI on the upregulation of ACE II is weakened, thus accelerating cognitive impairment. Moreover, ACEI use may be related to other mechanisms involved in AD pathogenesis, including antagonizing Ang II’s ability to inhibit acetylcholine release and regulate inflammation, which are characteristics of other neurodegenerative diseases. Currently, the clinical effect of ACEIs on stroke remains unknown.

ACEIs can improve cerebral blood flow, stabilize cognitive function in patients with MCI, and delay the decline of cognitive function in patients with hypertension. ACEIs can also inhibit the degradation of substance P, enkephalin, dynorphin, and neurotensin, which may subsequently enhance cognitive function and delay cognitive impairment in patients with hypertension. In summary, ACEIs can delay cognitive decline in elderly hypertensive patients with AD and reduce dementia in MCI patients.

## Diuretics

The exact effect of diuretics on cognitive function in the elderly has not been fully understood, primarily because previous studies used diuretics as concomitant antihypertensive drugs rather than as monotherapies. Most researchers believe that diuretics can reduce the incidence of cognitive decline in elderly patients with hypertension, and that different types of diuretics have varying effects on cognitive function [38]. In the Cache Country Cohort Study, antihypertensive drug use was associated with a reduced risk of AD (adjusted hazard ratio [AHR] = 0.77), among which thiazides (AHR = 0.7) and potassium sparing diuretics (AHR = 0.69) were associated with a similar reduced risk of AD (30%) [39]. These associations remained significant after adjusting for demographic data and cardiovascular risk factors. Some evidence suggests that blood potassium levels may also have an important effect on cognitive decline. Hypokalemia is related to a decrease of Aβ 42 in the cerebrospinal fluid [40]. Potassium supplementation can improve learning and memory impairment in rats with chronic cerebral hypoperfusion [41]. When potassium sparing and thiazide diuretics are used together, the incidence of dementia is significantly reduced, especially for people > 65 years of age who have high risk factors of dementia, present with a history of MCI, and carry the ApoE4 allele [42]. According to the retrospective analysis of the Ginkgo Evaluation of Memory Study [43], potassium sparing diuretics can protect cognitive function, as well as improve language and memory function, while loop and thiazide diuretics have no significant benefit for cognitive improvement. A recent comprehensive meta-analysis of more than 50,000 participants from 27 studies with individual patient data showed no association of diuretics and cognitive impairment or dementia in people > 65 years of age [10]. Although some of the included studies showed benefits of diuretics on cognitive function in hypertensive patients > 65 years of age, the results were difficult to interpret because of the heterogeneities in follow-up time, comparison groups, and outcomes [10].

There are few studies regarding role of diuretics on cognitive function in animal models of AD. At present, most studies have found that diuretics, especially potassium sparing diuretics, may delay the decline of cognitive function in elderly patients with hypertension. It is speculated that changes in serum potassium levels may be the underlying determinant. Potassium levels can impact cognitive decline. Hypokalemia is related to a decrease in Aβ (Aβ42) in the cerebrospinal fluid. Potassium supplementation can improve the learning and memory impairment of chronic cerebral hypoperfusion in rats, which may be related to the protective mechanism of potassium sparing diuretics.

## Angiotensin II receptor blockers (ARBs)

The renin-angiotensin-aldosterone system (RAAS) is closely associated with the pathogenesis of hypertension and lipids dysregulation [44]. Changes in the RAAS can affect also the pathogenesis of AD [29]. RAAS is mainly controlled by generating Ang II via direct vasoconstriction and reabsorption of sodium. Ang II is transformed from Ang I by ACE and is an important effector peptide in the RAAS, in that it exerts its biological activity by binding to the angiotensin (AT)1 and AT2 receptors [45].

In the Study on Combination and Diagnosis in the Elderly (SCOPE) [46], candesartan, an ARB drug, did not reduce the incidence of dementia. However, a subgroup analysis with extended follow-up showed that MMSE scores in the candesartan group decreased significantly. Subsequently, the Observational Study on Cognitive function And SBP Reduction (OSCAR) further showed consistent benefits of ARBs on cognitive function [47]. OSCAR is a large (n = 25,745) multi-ethnic longitudinal open trial, which aims to evaluate the cognitive functional decline and BP of patients aged > 50 years of age who have simple systolic hypertension and have been treated with Eprosartan (a highly selective AT1 receptor antagonist) for 6 months [47]. The initial results showed that MMSE scores were significantly increased compared to baseline after Eprosartan treatment (+ 0.8 points; P < 0.0001) [47]. In a subsequent study, [48] it was shown that, in addition to lowering BP, Eprosartan treatment reduced pulse pressure, suggesting that AT1 receptor antagonists may reduce arterial stiffness and contribute to cognitive improvement.

A number of studies have shown that ARBs can increase cerebral blood flow perfusion, reduce Aβ deposition, inhibit inflammatory responses, and promote nerve function recovery, all of which are neuroprotective [49-51]. Compared with ACEIs or other cardiovascular drugs, ARBs can significantly reduce the incidence of dementia and the progression of AD in a dose-dependent manner [52]. In hypertensive patients with AD, the ARB Telmisartan was associated with better cognitive improvement compared to amlodipine (a CCB), but with a similar reduction in BP [53]. Interestingly, previous meta-analyses showed that ARBs can be more effective at improving cognitive function than ACEIs, diuretics, and β-blockers in hypertensive patients without cerebrovascular disease, but the difference between the influence of ARBs and CCBs on cognitive function was not significant [54, 55]. Related animal experiments also showed that ARBs improved cognitive function in aged, stressed, and ischemic mice independent of lowering BP [56-58].

In summary, ARBs can increase cerebral blood flow perfusion, reduce Aβ accumulation, inhibit inflammatory reactions, and promote nerve function recovery. For patients with normal cognitive function or MCI, ARBs can reduce the levels of total and phosphorylated tau protein, increase the level of a β 1 - 42 in the cerebrospinal fluid, and reduce the risk of dementia. Moreover, ARBs can improve cerebral perfusion, reduce neurological dysfunction after cerebral ischemia, reduce inflammatory and oxidative stress markers, reduce the expression of low-density receptor and e-apolipoprotein, and increase the level of BDNF in the hippocampus. Because of their demonstrated protection against cognitive dysfunction, ARBs have been commonly prescribed for elderly hypertensive patients.

## Calcium Channel Blockers (CCBs)

The Europe (syst EUR) study was the first clinical randomized double-blind trial to study antihypertensive drugs, cognitive function, and dementia [58]. This study included patients aged ≥ 60 years of age without stroke history. The study showed that the incidence rate of vascular dementia and AD dementia decreased by 50% in patients receiving nitrendipine compared to those receiving placebo [58]. Other studies also confirmed that CCBs can prevent dementia and reduce the risk of occurrence and transformation of dementia [59-61], which may be related to neuroprotection in elderly patients with hypertension independent of BP lowering efficacy [62, 63]. In a meta-analysis of 13 studies, Angeli proposed that dihydropyridine showed a significant benefit in improving cognitive function in stroke patients, independent of lowering BP lowering, suggesting the possible neuroprotective effect of CCBs [64]. Pharmacologically, CCBs limit calcium entry by reducing the number of open calcium channels on the cell membrane. This mechanism of action is related to amyloidosis, because Aβ has been shown to destroy calcium homeostasis by inducing the formation of calcium permeable pores on the cell membrane, leading to neurodegeneration [65]. Moreover, CCBs may prevent cognitive impairment by correcting calcium levels, preventing calcium influx, and reducing nerve injury. Other studies showed that memory in stroke patients significantly improved at 3 months follow-up after nimodipine treatment in the acute phase of ischemic stroke [66]. According to the registration data of the Cochrane dementia and cognitive improvement group, nimodipine has certain benefits in the treatment of unclassified dementia, AD, vascular dementia, and mixed dementia [67]. In addition, nimodipine is well tolerated with few side effects, but its long-term anti-dementia effect needs to be confirmed [67]. Another randomized clinical trial (RCT) demonstrated that nimodipine can effectively treat subcortical vascular dementia due to cardiovascular protective effects [68]. Some studies confirmed that CCBs can reverse pathological changes in the microvascular structure in patients with essential hypertension, while β-blockers and diuretics do not have such effects [69, 70]. Finally, CCBs can play an additional protective role in the peripheral and central axes by improving structural damage and increasing blood vessel elasticity [71]. Long-term clinical trials are needed to validate the long-term benefits of CCBs on cognitive function in elderly patients with hypertension.

Accumulating evidence suggests that CCBs can reduce the incidence of cognitive impairment to varying degrees. The possible underlying mechanisms include restoring calcium homeostasis, preventing calcium influx, and correcting nerve injury associated with calcium influx. In addition, CCBs can reverse pathological changes in the microvascular structure associated with essential hypertension. They may also play a protective role in the peripheral and central nervous systems, which may improve cognitive function in elderly hypertensive patients.

## β-blockers

There is controversy in the literature regarding the effect of β-blockers on cognitive function [72]. One of the main limitations in the previous research is that, similar to diuretics, β-blockers are not commonly used as a monotherapy in the elderly population, but are often used in combination with other antihypertensive drugs. Animal studies have shown that carvedilol reduces levels of Aβ 1-40 and Aβ 1-42 in the brain, but does not improve cognitive function [73, 74]. In contrast, the use of selective β2-adrenergic receptor (β-2AR) antagonists can significantly worsen working memory, increase the burden of amyloid plaques, increase the level of a Aβ 1-42, and promote tau phosphorylation and deposition in the hippocampus [75]. It has been suggested that β-blockers can reduce the risk of AD in the elderly [76], but their neuroprotective effect is weaker than other antihypertensive drugs [77]. Another study including Asian male patients with hypertension found that β-blockers alone can reduce the risk of cognitive decline [78]. However, an earlier study suggested that β-blockers had little effect on cognitive improvement in elderly patients with hypertension [79]. Moreover, there are only a few studies that have investigated if the effect of β-blockers on cognitive function differs based on the drug’s characteristics, such as those that are selective or non-selective. Therefore, the exact influence of β-blockers on cognitive function in elderly patients with hypertension should be further elucidated in large-scale clinical studies.

## Clinical implications and conclusions

Hypertension has been associated with cognitive impairment in the elderly population. Cognitive decline and dementia lead to increased morbidity and mortality in elderly patients with hypertension. However, the findings previous studies regarding the association between BP, BPV, and antihypertensive drug use and the risk of cognitive impairment in elderly patients with hypertension have been inconsistent. Given the global burden of hypertension, the aging population, and the low quality of life associated with cognitive decline and/or dementia, a more comprehensive understanding of the association between hypertension and cognitive impairment is needed to better manage hypertension and reduce the risk of dementia. Based on the current evidence, the correlation of BP and risk of cognitive function seems to be U-shaped and dependent on age. Intensive BP control is generally not recommended, particularly for the oldest-old, in cases of increased risk of dementia. Increased BPV and characteristics of OH in the elderly increase the risk of cognitive decline. Current evidence does not support the use any one category of antihypertensive drug as a superior treatment strategy. There is evidence to support the use of any effective BP lowering drug class, including ARBs, ACEIs, CCBs, and aldosterone antagonists, to prevent cognitive decline in elderly patients with hypertension. The combination of two or more antihypertensive drugs is preferable to monotherapy because the combination confers cumulative and synergistic effects on BP control, stroke prevention, and possible cognitive impairment. More sensitive cognitive testing methods and longer follow-up testing is required, considering the complexity of hypertension in the elderly and the factors (such as age, gender, race, genetics, and mechanisms of action of different antihypertensive drugs) that may affect clinical outcomes. More RCTs should be performed to further explore how to better manage BP and to inform clinical use of more suitable antihypertensive drugs to promote cognitive health among the elderly. In this regard, more RCTs comparing the efficacy of antihypertensive drug use on cognitive function are needed to optimize BP management in the elderly who are at increased risk of dementia.
